# In Vitro Response of Two Strains of *Cordyceps javanica* to Six Chemical Pesticides

**DOI:** 10.3390/jof10120852

**Published:** 2024-12-10

**Authors:** Ruixia Mao, Xiaoxia Cai, Tengyu Wang, Ziyang Liu, Peixiang Xing, Guisen Zhang, Wenwen Zhou, Hongliang Diao, Ruiyan Ma

**Affiliations:** 1 College of Plant Protection, Shanxi Agricultural University, Taiyuan 030031, China; 18335906241@163.com (R.M.);; 2 State Key Laboratory of Integrative Sustainable Dryland Agriculture (In Preparation), Shanxi Agricultural University, Taiyuan 030031, China

**Keywords:** chemical pesticides, *Cordyceps javanica*, compatibility, growth inhibition, biological control

## Abstract

To determine the compatibility of two new biocontrol fungi with common chemical pesticides, this study examined the effects of three insecticides, namely, avermectin, imidacloprid, and acetamiprid, and three fungicides, namely, chlorogenonil, boscalid, and kasugamycin, on the mycelial growth and spore germination of *Cordyceps javanica* strains IF-1106 and IJ-tg19. The insecticidal effects of mixed insecticides or fungicides with good compatibility with *C. javanica* IJ-tg19 against *Myzus persicae* were tested. The results showed that the six chemical pesticides exerted different degrees of inhibition on the mycelial growth of both *C. javanica* strains, with an obvious dose-dependent effect. The inhibitory effect of chlorothalonil on the mycelial growth of IF-1106 and IJ-tg19 was greater than 75%. Different kinds and concentrations of chemical pesticides had significant effects on spore germination. Among them, acetamiprid had little inhibitory effect on *C. javanica* spores. Therefore, the two *C. javanica* strains exhibited good compatibility with the insecticide acetamiprid and had some compatibility with avermectin and imidacloprid. Among the fungicides, the compatibility of the two strains of biocontrol fungi was the best with kasugamycin, followed by boscalid, while their compatibility with chlorothalonil showed the least compatibility. The median lethal time (LT_50_) of five concentrations of *C. javanica* IJ-tg19 (1 × 10^3^, 1 × 10^4^, 1 × 10^5^, 1 × 10^6^, and 1 × 10^7^ spore/mL) mixed with acetamiprid against *M. persicae* were 5.28, 4.56, 3.80, 2.73, and 2.13 days, respectively, and the insecticidal rate was higher than that of fungus treatment alone (5.19, 4.59, 4.05, 3.32, and 2.94 days, respectively) or chemical pesticide treatment (5.36 days). This study provides data support and a theoretical basis for reducing the use of chemical pesticides, improving the efficiency of *C. javanica*-based insecticides, and optimizing the synergistic use of fungi and chemical pesticides.

## 1. Introduction

*Myzus persicae* (Sulzer) (Hemiptera: Aphididae) [1], an important pest affecting peach trees, plum trees, tobacco, sugar beets, and cruciferous vegetables, is widely distributed around the world [2]. In addition to causing direct damage to crops, *M. persicae* may indirectly damage crops through the transmission of plant viral diseases. At present, chemical control is the main means used to control peach aphids, but the frequent and irregular application of chemical insecticides leads to the emergence of resistance, which increases the difficulty of control. Therefore, it is imperative to develop safe, efficient, and ecologically sustainable green prevention and control technologies.

Entomopathogenic fungi (EPF) are an important biological control resource for the control of insect pests. The wide application of fungal insecticides that utilize EPF as active substances is of great significance in the continuous control of insect pests, the protection of beneficial organisms, and the maintenance of ecological balance. As an important biocontrol fungus, *Cordyceps javanica* (Friederichs & Bally) (Hypocreales: Cordycipitaceae) can infect various instars of *Bemisia tabaci* (Gennadius) (Hemiptera: Aleyrodidae), and can effectively control agricultural pests such as *Plutella xylostella* (Linnaeus) (Lepidoptera: Plutellidae) and *M. persicae* [3,4,5,6]. Fungal insecticidal preparations with *C. javanica* as the active ingredient, such as PFR-97, which was jointly developed by the United States and Mexico, and PreFeRal, which was jointly developed by many European countries, have been successfully applied to the sustainable control of whitefly [7]. In China, *C. javanica* has also been registered for the control of *B. tabaci* and *Spodoptera litura* (Fabricius) (Lepidoptera: Noctuidae) [8].

Fungal insecticides have the advantages of non-resistance and environmental friendliness, but their wide application still faces many challenges. Fungal insecticides utilize live spores as active ingredients, and their efficacy is readily affected by synthetic chemicals applied to crops. This sensitivity to chemical pesticides leads to a limited synergistic application effect, which is an important problem in the utilization of fungal insecticides. Research has shown that the inhibition effect of different kinds of chemical pesticides on biocontrol fungi varies widely. A previous study found that the compatibility of common fungicides (difenoconazole, propiconazole, trifloxystrobin, azoxystrobin, carbendazim, and hexaconazole) with *Metarhizium anisopliae* (Sorokin) (Hypocreales: Clavicipitaceae) was low, and the spore germination rate decreased by more than 50% at 48 h, indicating that this biocontrol fungus could not be mixed with these fungicides [9,10]. Herbicides such as pendimethalin, 2,4-D, trifluralin, phenmedipham, metolachlor, and chloridazon all have an obvious inhibition effect on EPF. However, lenacil, imazapic, bentazon, and glyphosate exhibit some level of compatibility with EPF. Insecticides have good compatibility with a variety of EPF. For example, previous research demonstrated that the inhibitory rates of a sublethal concentration of fenvalerate on the spore production and mycelial growth of *M. anisopliae* were 38.63% and 25.13%, respectively, and fenvalerate had no effect on spore germination [11]. The inhibition rates of spinosad and imidacloprid on the mycelial growth of *M. anisopliae* at relatively high concentrations were only 11.41% and 14.44%, respectively. Therefore, spinosad and imidacloprid can be mixed with *M. anisopliae* at certain concentrations [9]. Bifenthrin exhibits good compatibility with *Beauveria bassiana* (Balsamo-Crivelli) (Hypocreales: Cordycipitaceae), with no significant inhibitory effect on spore germination or mycelial growth [12]. The negative effects of chemical pesticides on EPF are generally manifested as fungicides > herbicides > insecticides.

To realize the combined application of fungal insecticides and chemical pesticides to improve the efficiency of pest control, it is necessary to clarify the compatibility of EPF and common chemical pesticides. Many reported results have demonstrated the feasibility of this research direction. For example, the combination of *M. anisopliae* and chlorantraniliprole showed an apparent synergic insecticidal effect on *Locusta migratoria* (Linnaeus) (Orthoptera: Acrididae). The mortality rate of *L. migratoria* after treatment with 2.0 × 10^8^ spores/mL + 1 mg/L chlorantraniliprole reached more than 80%, while the mortality rate following treatment with 20 mg/L chlorantraniliprole was only 63.3% [13]. The combined application of diflubenzuron with *M. anisopliae* and *B. bassiana* can improve the control efficiency of *Culex pipiens* (Linnaeus) (Diptera: Culicidae); although *M. anisopliae* is more suitable for this application than *B. bassiana* [14], there are also chemical insecticides used in conjunction with *B. bassiana*. Compared with insecticides alone, the application of the 10% recommended concentration of beta-cypermethrin combined with 1 × 10^7^ spores/mL of *B. bassiana* PfBb resulted in a higher cumulative death rate of 93.49% for *Phauda flammans* (Walker) (Lepidoptera: Phaudidae) [15]. In addition, the application of β-cyfluthrin also significantly increased the mortality of *Alphitobius diaperinus* (Panzer) (Coleoptera: Tenebrionidae) larvae infected with *B. bassiana*. However, the combination of imidacloprid, spinosad, and *B. bassiana* showed no notable synergistic effect on the control of *A. diaperinus* larvae [16]. Although the application of fungal and chemical insecticides can be reduced to a certain extent, it is necessary to continue to find tacit partners to expand their synergies.

Various kinds and concentrations of chemical pesticides have different effects on EPF, and the same pesticide may have varying effects on different strains of the same fungus. The compatibility between pesticides and EPF follows no reliable rule or model. In this study, two potential biocontrol fungi, *C. javanica* IF-1106 and *C. javanica* IJ-tg19, were selected as the research objects, and three insecticides for the control of insects with piercing–sucking mouthparts in addition to three common fungicides were selected to conduct compatibility tests between the strains and chemical pesticides. The combined virulence of the fungi and chemical control agents was verified on the basis of compatibility. The aim of this study was to clarify the inhibitory activity and inhibitory dose of several chemical pesticides on the tested strains, and to provide a theoretical foundation and data support for the collaborative application of fungal insecticides and chemical pesticides based on the above strains.

## 2. Materials and Methods

### 2.1. Culture of Fungi

The *Cordyceps javanica* (Frieder. & Bally) Kepler, B. Shrestha & Spatafora strains IF-1106 and IJ-tg19 were provided by the Biosafety and BioControl Laboratory, Shanxi Agricultural University. *C. javanica* was obtained from the original sample using a sterile inoculation ring and inoculated onto the surface of a potato dextrose agar (PDA) medium (Qingdao Hope Bio-Technology Co., Ltd., Qingdao, China) using the streak plate method. The whole process was conducted on a super-clean workbench to ensure sterility. After inoculation, the PDA plate was placed upside down in a constant temperature incubator (Shanghai Boxun Medical Biological Instrutent Corp., Shanghai, China) at 25 °C and incubated for 10 days.

### 2.2. Chemical Pesticides

Six common chemical pesticides were selected, comprising three insecticides and three fungicides. The sources of the pesticides are listed in Table 1.

### 2.3. Preparation of Treated Medium

Each pesticide was diluted in water according to multiples of 1, 5, 25, 125, and 625, and an equal volume of sterile water was added as a blank control. A pipette was used to inject 1 mL of pesticide from a centrifuge tube into a Petri dish, and three drops of 25% lactic acid were added to each Petri dish to inhibit bacterial growth. Finally, 9 mL of the heated melted PDA medium was added to the treated Petri dish and gently shaken for mixing, and the treated medium was obtained after solidification.

### 2.4. Determination of Spore Germination Rates

Spore germination assays were conducted based on Ma’s method, with several modifications implemented to suit the specific requirements of this study [17]. First, 50 μL of a spore suspension was removed with a pipette, injected onto the center of the pesticide-treated medium, and then evenly spread across the medium using a triangular rod in a circular pattern. After sealing the medium, the culture was placed in an incubator at a constant temperature of 25 °C for 48 h under dark conditions, after which plates with 30–300 colonies were selected for counting. The colony diameter of each treatment was obtained as the mean of three biological replicates. The inhibition rate of spore germination was calculated as follows:

Germination rate (%) = Spore germination number/300 × 100,(1)

### 2.5. Strain Growth Rate Assay

We referred to Zhang’s method and made some improvements [18]. The *C. javanica* strains IF-1106 and IJ-tg19 were activated on the PDA medium using the plate scribing method and then cultured in an incubator at 25 °C in the dark for 4 d to obtain single colonies of an appropriate size. Under aseptic conditions, a sterilized 7 mm hole punch was used to obtain circular samples of an equal area from the edges of the fungal colonies, and a sterilized inoculation ring was used to inoculate the fungi in the centers of the Petri dishes containing the treated medium, with the mycelium side facing down. The dishes were placed in an incubator at a constant temperature of 25 °C under dark conditions.

After 2 d of culture, the diameter of each colony was measured twice using the cross method and averaged, and the colony diameter was recorded each day from the third day onward. The colony diameters of each treatment were obtained by averaging the results of three replicates. The inhibition ratio was calculated as follows:

Inhibition ratio (%) = (Control colony diameter − Colony diameter of treatment group)/Control colony diameter × 100,(2)

### 2.6. Effects of C. javanica Mixed with Low Doses of Acetamiprid and Kasugamycin on the Insecticidal Activity of M. persicae

#### 2.6.1. Test Insect Source

The aphids, *M. persicae*, were provided by the Research Group of Biosafety and BioControl, the College of Plant Protection, Shanxi Agricultural University. After several generations of stable breeding on pepper leaves, the newly molted adult insects were subjected to bioassay screening. The feeding material was grown naturally without spraying pesticides.

#### 2.6.2. Bioassay

The spore suspensions of *C. javanica* were set at five concentrations of 1 × 10^3^, 1 × 10^4^, 1 × 10^5^, 1 × 10^6^, and 1 × 10^7^ spores/mL. The process was configured using an aqueous solution containing 0.1% Tween-80 in order to disperse the spores evenly in the liquid and avoid settling. Acetamiprid and kasugamycin were diluted 625 times, and the five concentrations of *C. javanica* were mixed with low doses of acetamiprid and kasugamycin. The test method was based on the biodetermination of the pathogenicity of *C. javanica* against *M. persicae* described by Xing [19], with appropriate modifications. The specific methods were as follows: The self-planted pepper leaves were collected and washed under running water, disinfected with 75% alcohol spray, and cut into appropriately sized rounds for placing in 60 mm Petri dishes. The Petri dishes were moisturized with a water–agar medium and the leaves were placed in the dishes. Fifteen newly molted peach aphids of the same age were added to the leaves in each dish. After a short wait for the peach aphids to begin feeding, spore suspensions of different concentrations (1 × 10^3^, 1 × 10^4^, 1 × 10^5^, 1 × 10^6^, and 1 × 10^7^ spores/mL) were evenly sprayed into each dish using a throat sprayer (spraying 20 times). To prevent the insects from escaping, the dish cover was covered with 75% alcohol to disinfect the newspaper, and the Petri dish was sealed with a sealing film. Each dish contained 15 aphids, and the test was repeated four times. Treatment with 0.1% Tween-80 solution spray was utilized as a blank control. After spray inoculation, the insects were fed in a light incubator (25 °C, 12 h light/12 h dark). The number of *M. persicae* was recorded each day, and newborn aphids were removed and observed for 7 days. Dead *M. persicae* were cultured on PDA to determine whether they were infected by *C. javanica* IJ-tg19.

#### 2.6.3. Data Analysis

SPSS 27 software (International Business Machines Corporation, Armonk, NY, USA) was used to test the normal distribution of the data, and a one-way analysis of variance (ANOVA) was performed to compare the difference in the spore germination rate between the two EPF strains at different concentrations. The significance of differences was analyzed using Tukey’s method. The column chart of the germination rate was designed in GraphPad Prism 10 (Graphpad software, San Diego, CA, USA). The Kaplan–Meier method in Origin 2021 (OriginLab, Northampton, MA, USA) was employed to analyze the survival curve of peach aphids exposed to each fungus–drug combination.

## 3. Results

### 3.1. Effects of Three Fungicides on the Spore Germination of Two C. javanica Strains

**Figure 1:**
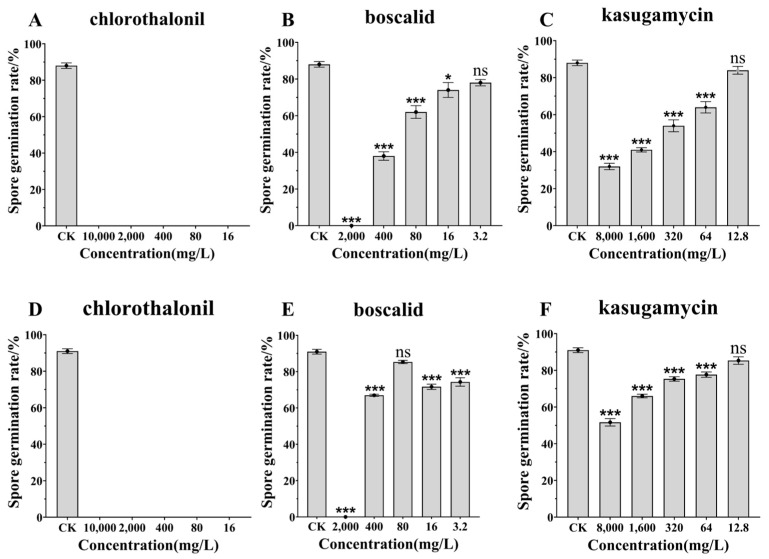
Effects of three fungicides on spore germination of *Cordyceps javanica* IJ-tg19 (**A**–**C**) and IF-1106 (**D**–**F**). Note: ns means no difference, * significance to *p* < 0.05, and *** significance to *p* < 0.001. CK: sterilized distilled water. Data points represent the mean ± the standard error of the mean (SEM).

Compared to the control group, each tested fungicide exerted different degrees of inhibition on the conidial germination of the two strains of biocontrol fungi. The data results conformed to a normal distribution. Among the tested fungicides, 75% chlorothalonil had the greatest effect on the inhibition of the conidial germination of *C. javanica* strains IJ-tg19 (Figure 1A) and IF-1106 (Figure 1D). The conidial germination of the two strains was completely inhibited and the conidial germination rate was close to 0%. Under normal concentrations, 50% boscalid completely inhibited the conidial germination of strains IJ-tg19 and IF-1106, but the effect gradually decreased with the dilution ratio (Figure 1B, IJ-tg19: F = 163.856, df = 17, *p* < 0.001; Figure 1E, IF-1106: F = 726.657, df = 17, *p* < 0.001). On the whole, the inhibitory effect of 2% kasugamycin decreased with the dilution ratio. At ratios greater than 625, 2% kasugamycin had little effect on the spore germination of the two strains (Figure 1C, IJ-tg19: F = 99.998, df = 17, *p* < 0.001; Figure 1F, IF-1106: F = 90.971, df = 17, *p* < 0.001).

### 3.2. Effects of Three Insecticides on the Spore Germination of Two C. javanica Strains

**Figure 2:**
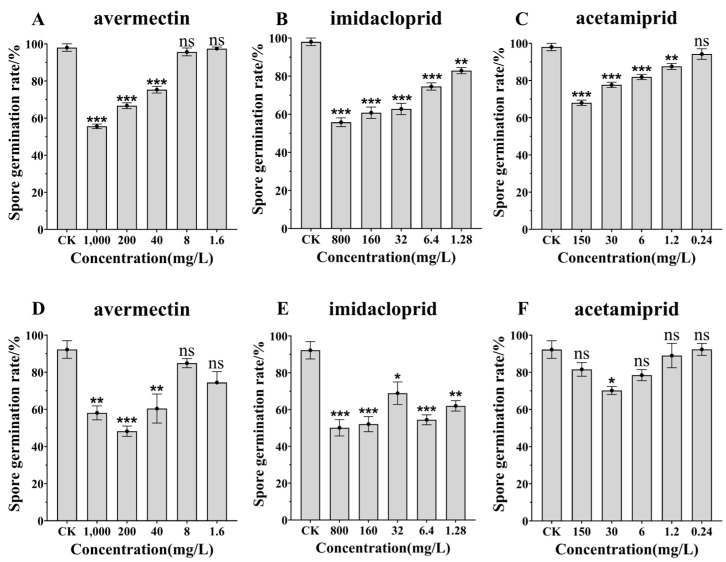
Effects of three insecticides on spore germination of *Cordyceps javanica* IJ-tg19 (**A**–**C**) and IF-1106 (**D**–**F**). Note: ns means no difference, * significance to *p* < 0.05, ** significance to *p* < 0.01, and *** significance to *p* < 0.001. CK: sterilized distilled water. Data points represent the mean ± the standard error of the mean (SEM).

The three tested insecticides had significant effects on the spore germination rates of *C. javanica* strains IF-1106 and IJ-tg19 under different dilution ratios, where the lower the dilution ratio, the greater the effect. The data distribution is consistent with the normal distribution. The effects of the tested insecticides on the two strains of biocontrol fungi were in the following order: 25% imidacloprid > 2% avermectin > 60% acetamiprid. The insecticide with 25% imidacloprid was highly toxic to *C. javanica* strains IF-1106 and IJ-tg19 and inhibited spore germination even at high dilution ratios (Figure 2B, IJ-tg19: F = 49.376, df = 17, *p* < 0.001; Figure 2E, IF-1106: F = 13.103, df = 17, *p* = 0.016). The inhibition rate of 2% avermectin on the spore germination of the two strains reached more than 40% under the conventional concentration (Figure 2A, IJ-tg19: F = 140.219, df = 17, *p* < 0.001; Figure 2D, IF-1106: F = 11.639, df = 17, *p* < 0.001). The 60% acetamiprid had the least effect on the spore germination of the two strains, with the germination rates being greater than 60% under different dilutions, and the spore germination rate increased with the dilution (Figure 2C, IJ-tg19: F = 40.289, df = 17, *p* < 0.001; Figure 2F, IF-1106: F = 4.434, df = 17, *p* < 0.001).

### 3.3. The Effects of Pesticides on the Mycelial Growth of Two C. javanica Strains

The effects of the six pesticides on the mycelial growth of the two strains are shown in Table 2. The six tested pesticides inhibited the mycelial growth of strains IJ-tg19 and IF-1106, and the degree of inhibition varied. In general, the fungicides exerted stronger inhibitory effects on mycelial growth than the insecticides. The compatibility between 75% chlorothalonil and the two strains was the lowest, and the inhibition rate of the mycelial growth of the two strains reached more than 90%. With the increase in dilution, the inhibition rates of 50% boscalid and 2% kasugamycin on the mycelial growth of strains IF-1106 and IJ-tg19 showed decreasing trends. The inhibitory effects of the three insecticides on the mycelial growth of the two strains were weak, no more than 30%, and the inhibitory effects decreased with the increase in the dilution ratio.

### 3.4. Comprehensive Evaluation of Compatibility Between Six Pesticides and Two Strains of C. javanica

The effects of six commonly used pesticide preparations on the mycelial growth and spore germination of two strains of biocontrol fungi were analyzed, and the results showed that the insecticides had greater compatibility with the two strains of EPF than the fungicides. The three fungicides inhibited the growth of both strains of EPF, and the compatibility was poor. The three insecticides showed inhibitory effects on the two strains of EPF during the high-concentration stage, and the inhibitory effects gradually disappeared when the dilution ratio of the insecticides exceeded 625 times.

### 3.5. Pathogenicity of C. javanica Combined with Chemical Pesticides on M. persicae

**Figure 3:**
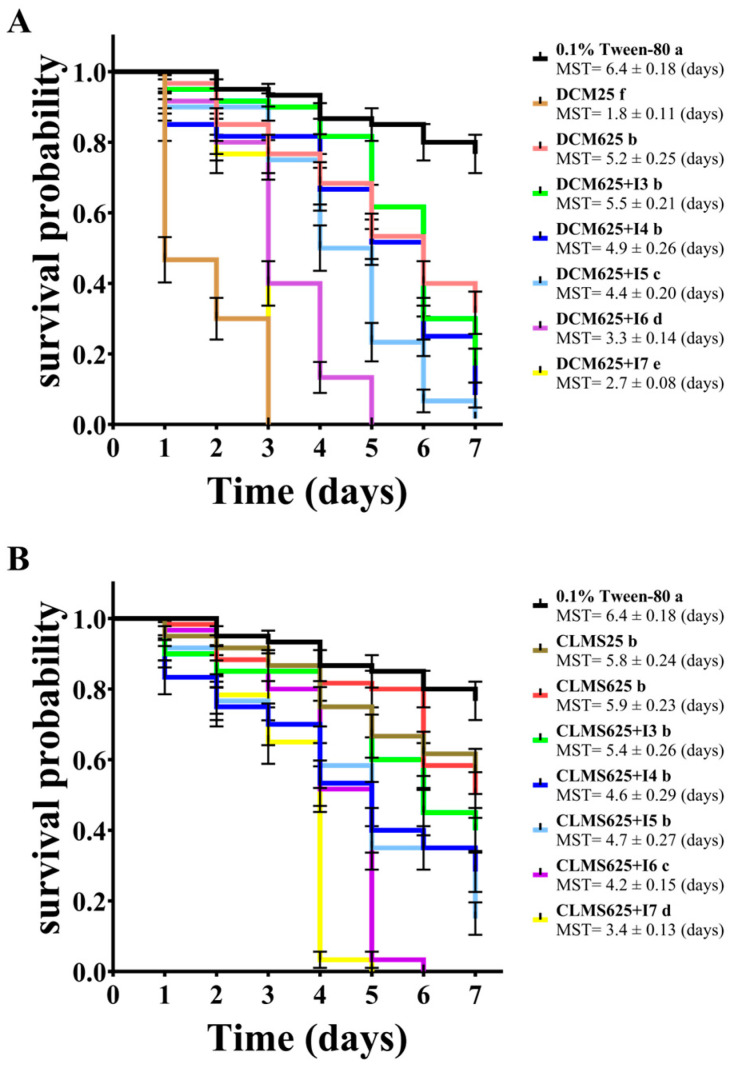
Survival function induced by *Cordyceps javanica* IJ-tg19 mixed with acetamiprid (**A**) and kasugamycin (**B**) in *Myzus persicae*. Note: CLMS means kasugamycin, DCM means acetamiprid, and 25 and 625 mean dilutions. I means *C. javanica* IJ-tg19; the numbers 3, 4, 5, 6, and 7 are spore concentrations of *C. javanica* IJ-tg19: 1 × 10^3^ spores/mL, 1 × 10^4^ spores/mL, 1 × 10^5^ spores/mL, 1 × 10^6^ spores/mL, and 1 × 10^7^ spores/mL. Lowercase letters indicate differences at the 0.05 significance level in survival probabilities between groups, not differences in the median survival time (MST).

The compatibility of the insecticide acetamiprid, the fungicide kasugamycin, and *C. javanica* IJ-tg19 when applied in combination or alone and their virulence against *M. persicae* were determined under indoor conditions (Table 3). The results showed that the insecticidal effect of EPF combined with chemical pesticides on *M. persicae* was obviously higher than those of EPF or insecticides alone. Among the treatments, under the condition of a dilution ratio of 625, the toxicity of acetamiprid mixed with five concentrations of *C. javanica* strain IJ-tg19 to *M. persicae* was higher than that of each agent alone, and the synergism was significant. The median lethal time (LT_50_) values for *M. persicae* were lower than those of *C. javanica* strain IJ-tg19 alone and the pesticide alone (5.36 days), and there were significant differences in the survival analysis (Figure 3A). Under the condition of a dilution ratio of 625, there was no significant difference between the LT_50_ of kasugamycin mixed with *C. javanica* strain IJ-tg19 and *C. javanica* alone. Therefore, the mixed application of the low-dose insecticide acetamiprid and *C. javanica* IJ-tg19 has a synergistic effect, which is beneficial for sustainable pest control using *C. javanica* in the field. In addition, the low-dose fungicide kasugamycin has good compatibility with *C. javanica* and does not affect the lethal effect of *C. javanica* strain IJ-tg19 when mixed (Figure 3B).

## 4. Discussion

In this study, the effects of six chemical insecticides and fungicides on the mycelial growth and spore germination of *C. javanica* strains IF-1106 and IJ-tg19 were measured. Compared with the control group, the three chemical fungicides exhibited strong inhibitory effects on the mycelial growth and spore germination of the two strains of biocontrol fungi, and the inhibitory effect of chlorogenonil was the most significant. Relatively speaking, the three chemical insecticides had little effect on the two strains of biocontrol fungi, and their compatibility was obviously better than that of fungicides, demonstrating the potential of these insecticides in compounding the effects of and synergistic application with EPF.

*C. javanica* can be employed in combination with appropriate pesticides to enhance the pest control effect and reduce the use of pesticides. In this study, the insecticidal effect of 625-fold-diluted acetamiprid combined with *C. javanica* strain IJ-tg19 was significantly better than that of acetamidine alone, and the overall dose was reduced, indicating a significant synergistic effect between the two. Acetamiprid can also be utilized in combination with *B. bassiana*, and its control effect on *B. tabaci* can reach 85.5% [20]. The combination of EPF and chemical insecticides has become an important method to reduce pesticide application and increase efficiency. For example, the combination of gamma-cyhalothrinand with *B. bassiana* LPSc 1067 can significantly increase the mortality of *Rachiplusia nu* larvae at 25% of the recommended field dose, showing a synergic effect [21]. Similarly, adding an appropriate amount of avermectin can reduce the amount of avermectin necessary and improve the control effect of *B. bassiana*. The mixed application of sublethal doses of imidacloprid and *B. bassiana* to *Leptinotarsa decemlineata* larvae also showed a synergistic effect in previous research, especially when imidacloprid was applied for 24 h followed by spraying with *B. bassiana* [22]. It is unclear why the combination of low concentrations of insecticides and EPF increases their effectiveness. Burges believes that low doses of chemical insecticides weaken insects’ resistance to fungi. It has also been pointed out that low concentrations of chemical insecticides stimulate the physiological stress response of pests or change their behavior, increasing the likelihood that EPF will invade hosts [23,24,25,26].

The results of this study showed that the sensitivity of *C. javanica* to chemical pesticides was affected by different pesticide species and mechanisms of action. The three insecticides inhibited the spore germination and mycelial growth of *C. javanica* at high concentrations. Although the inhibitory activity of avermectin on fungi is unclear, avermectin can kill drug-resistant bacteria such as *Mycobacterium tuberculosis* and *M.ulcerans* [27]. High concentrations of acetamiprid inhibited the spore germination and mycelial growth of *M. anisopliae* [28] and reduced the production of its insecticidal toxin, although it did not significantly affect the infection capacity of *M. anisopliae* [29]. Imidacloprid does affect fungal spore growth [30], but the specific inhibitory mechanism remains unclear. Compared with insecticides, fungicides inhibit EPF more significantly, and the mechanism is relatively clear. Fungicides mainly inhibit fungal growth via destroying the activity of fungal metabolic enzymes, but this mechanism has little inhibitory effect on *B. bassiana* [31], which may be due to differences between strains. In contrast, *C. javanica* was less sensitive to both boscalid and kasugamycin. Boscalid inhibits the respiratory metabolism of fungal spores, while mycelial energy is mainly obtained from the outside world and does not depend on respiration [32]. Kasugamycin interferes with fungal protein synthesis, but this interference is incomplete, and normal protein synthesis still occurs [33]. These may be some of the reasons for the low inhibitory effect of kasugamycin on *C. javanica*. Although the above bactericides exerted obvious inhibitory effects on the tested strains, it is still possible to improve their compatibility. Industrial-grade boscalid inhibits fungal growth, but its preparation product Cantus does not affect the growth of *B. bassiana* or its pathogenicity against *B. tabaci* [34]. Noticeably, the inhibitory effect of fungicides on EPF can be limited to some extent by improving their formulation. In addition, to ensure the efficacy of EPF, a certain interval should be maintained between the field application of EPF and chemical fungicides.

Fungal insecticides offer environmental compatibility, safety for humans and livestock, and a reduced risk of pest resistance. However, their practical effectiveness is hampered by the prolonged infection period and high resistance in healthy pests, resulting in delayed efficacy. To address this, low concentrations of chemical insecticides can be used to weaken pest defenses, thus facilitating fungal invasion and improving performance. In addition, the combination of fungal insecticides and chemical fungicides can simultaneously prevent pests and diseases, reducing the workload of control. The collaborative application of fungal insecticides and chemical pesticides is an effective way to reduce application and increase efficiency, which can not only make up for some obvious shortcomings of fungal insecticides, but is also a reliable choice between green production and effective control. Our research and that of many scholars supports this collaborative approach, which leverages the strengths of both, showing promising progress in agricultural pest control practices.

## 5. Conclusions

This study determined the compatibility of *C. javanica* strains IJ-tg19 and IF-1106 with several common chemical insecticides/fungicides. For the fungus–drug combinations with poor compatibility, the application interval and method should be clearly defined during collaborative application, and for the fungus–drug combinations with good compatibility, the synergistic mechanism should be further clarified. In addition, various EPF have different sensitivities to different agents. The type and dose of agents also affect the tolerance of EPF, and the toxicological target mechanism needs to be further clarified. The development of engineered strains with high tolerance to agents and the reduction in the susceptibility of EPF through the targeted formulation of agents to increase the resistance of strains to chemical agents are also foreseeable possible approaches.

## Figures and Tables

**Table 1:** The type, name, dosage form, recommended dosage, and manufacturer of the vegetable pesticide tested.

Active Ingredient		Dosage Form	Control Object	Recommended Dosage in the Field (g/L)	Manufacturer
Fungicides	75% chlorothalonil	Wettable powder	Cucumber powdery mildew	10	Shandong Xinxing Pesticide Co., Ltd., Qingzhou, China
	50% boscalid	Water-dispersible granule	Tomato early blight	2	Shaanxi Huarong Kaiwei Biological Co., Ltd., Xi’an, China
	2% kasugamycin	Water aqua	Tomato leaf mold	8	Jiangmen City Plant Protection Co., Ltd., Jiangmen, China
Insecticides	2% avermectin	Microcapsule suspension agent	Whitefly	1	Hebei Weiyuan Biochemical Pesticide Co., Ltd., Shijiazhuang, China
	25% imidacloprid	Wettable powder	Aphid	0.8	Shandong United Pesticide Industry Co., Ltd., Jinan, China
	60% acetamiprid	Wettable powder	Aphid	0.15	Shaanxi Xiannong Biotechnology Co., Ltd., Xi’an, China

**Table 2:** Inhibition rate of six pesticides on mycelial growth of two strains at 9th day.

Strains	Dilution Ratio	Average Rejection Rate/%					
		Fungicides			Insecticides		
		Chlorothalonil	Kasugamycin	Boscalid	Avermectin	Imidacloprid	Acetamiprid
IJ-tg19	1	100.00 ± 0.00	16.13 ± 5.59	35.48 ± 1.86	28.00 ± 1.39	29.20 ± 0.80	19.20 ± 0.80
	5	25.81 ± 1.86	15.05 ± 2.84	18.28 ± 2.84	16.80 ± 2.12	24.00 ± 2.88	11.20 ± 2.88
	25	18.28 ± 2.85	12.90 ± 1.86	16.13 ± 1.86	16.00 ± 1.38	22.40 ± 1.60	7.80 ± 2.12
	125	16.13 ± 0.52	9.68 ± 1.86	14.36 ± 1.63	5.60 ± 2.88	20.00 ± 0.80	3.73 ± 0.56
	625	6.45 ± 3.23	3.23 ± 1.86	12.90 ± 1.86	4.00 ± 1.39	7.20 ± 2.11	2.40 ± 0.80
IF-1106	1	95.10 ± 0.98	32.35 ± 3.40	32.35 ± 4.49	20.33 ± 3.12	19.39 ± 3.40	18.84 ± 1.98
	5	31.37 ± 3.53	30.39 ± 9.95	27.45 ± 4.27	15.15 ± 9.04	11.72 ± 3.38	11.78 ± 3.35
	25	23.53 ± 6.12	26.47 ± 3.39	22.55 ± 3.53	10.09 ± 1.38	10.25 ± 4.75	8.53 ± 3.81
	125	19.61 ± 4.27	14.71 ± 1.70	20.59 ± 3.40	5.54 ± 3.60	8.14 ± 2.11	3.54 ± 0.49
	625	17.65 ± 8.82	8.17 ± 2.94	8.82 ± 3.39	1.26 ± 2.65	4.51 ± 2.81	3.44 ± 1.29

**Table 3:** The toxicity to *Myzus persicae* of separately using pesticides and *C. javanica* and their combined use.

Concentration of *C. javanica* (Spores/mL)	Corrected Mortality (%) at the 7th Day (Mean ± SE)			*C. javanica*		+DCM625		+CLMS625	
				LT_50_ (Days)	95% Confidence Interval	LT_50_ (Days)	95% Confidence Interval	LT_50_ (Days)	95% Confidence Interval
	*C. javanica*	+DCM625	+CLMS625						
0	-	68.33 ± 5.69	50.00 ± 14.01	-	-	5.36	5.04~5.73	7.08	6.53~7.85
10^3^	73.33 ± 17.43 b	83.33 ± 16.67 a	60.00 ± 4.71 c	5.19	4.91~5.49	5.28	4.75~5.96	5.99	5.56~6.53
10^4^	86.67 ± 11.22 b	91.67 ± 8.33 a	71.67 ± 12.58 c	4.59	3.42~4.85	4.56	3.82~5.45	4.54	4.18~4.94
10^5^	96.67 ± 3.33 a	98.33 ± 1.67 a	85.00 ± 5.69 b	4.05	3.44~4.69	3.80	3.33~4.26	4.40	4.12~4.69
10^6^	100.00 ± 0.00 a	100.00 ± 0.00 a	100.00 ± 0.00 a	3.32	2.98~3.64	2.73	2.58~2.88	3.61	3.04~4.21
10^7^	100.00 ± 0.00 a	100.00 ± 0.00 a	100.00 ± 0.00 a	2.94	2.46~3.37	2.13	1.70~2.66	2.83	2.26~3.44

Note: lowercase letters indicate differences among treatments at the 0.05 significance level.

## Data Availability

The original contributions presented in this study are included in the article. Further inquiries can be directed to the corresponding authors.
